# Long-term exposure to moderate noise induces neural plasticity in the infant rat primary auditory cortex

**DOI:** 10.1080/19768354.2019.1643782

**Published:** 2019-07-19

**Authors:** Chenchen Xia, Manli Yin, Ping Pan, Fanghao Fang, You Zhou, Yonghua Ji

**Affiliations:** a Laboratory of Neuropharmacology and Neurotoxicology, Shanghai University, Shanghai, People’s Republic of China; b Department of Otolaryngology-Head and Neck Surgery, Ninth People’s Hospital, Shanghai Jiaotong University School of Medicine, Shanghai, People’s Republic of China; c Ear Institute, Shanghai Jiaotong University School of Medicine, Shanghai, People’s Republic of China; d Shanghai Key Laboratory of Translational Medicine on Ear and Nose Diseases, Shanghai, People’s Republic of China

**Keywords:** Noise exposure, hearing threshold, LFP oscillation, A1 cortex, glutamate receptor

## Abstract

Previous studies have reported that rearing infant rat pups in continuous moderate-level noise delayed the formation of topographic representational order and the refinement of response selectivity in the primary auditory (A1) cortex. The present study further verified that exposure to long-term moderate-intensity white noise (70 dB sound pressure level) from postnatal day (P) 12 to P30 elevated the hearing thresholds of infant rats. Compared with age-matched control rats, noise exposure (NE) rats had elevated hearing thresholds ranging from low to high frequencies, accompanied by decreased amplitudes and increased latencies of the two initial auditory brainstem response waves. The power of raw local field potential oscillations and high-frequency *β* oscillation in the A1 cortex of NE rats were larger, whereas the power of high-frequency *γ* oscillation was smaller than that of control rats. In addition, the expression levels of five glutamate receptor (GluR) subunits in the A1 cortex of NE rats were decreased with laminar specificity. These results suggest that the altered neural excitability and decreased GluR expression may underlie the delay of functional maturation in the A1 cortex, and may have implications for the treatment of hearing impairment induced by environmental noise.

## 1. Introduction

Noise pollution has been linked to a wide variety of adverse effects and has risen to become a severe public health problem in recent decades (Stansfeld and Matheson 2003; Basner et al. 2014). The adverse hearing effects from passive noise are dependent on several factors, including duration, frequency and intensity of exposure, as well as developmental age (van Kamp and Davies 2013). Normally, sounds with a sound pressure level (SPL) >105 dB are defined as traumatic noise, and those with an SPL level >80 dB are considered to be threatening noise (Eggermont 2017). Numerous studies in humans and animals have revealed that higher-intensity noise exposure can lead to permanent or temporary sensorineural hearing impairment from the peripheral to central auditory system. Excessive noise exposure (NE) can irreversibly damage the cochlea, increase the threshold of hearing sensitivity, weaken the time coding of auditory signals (Pourbakht and Yamasoba 2003; Chen et al. 2019; Frye et al. 2018), and dissimilate neural coding processing in the cochlear nucleus, inferior colliculus, and auditory cortex (Willott and Lu 1982; Kaltenbach et al. 2000). Recently, an ‘assumed safe’ noise with a sound level <80 dB SPL has been well noticed, because most people are exposed to this kind of non-traumatic environmental noise during daily life, rather than high-intensity noise (Eggermont 2017). Studies performed in adult cats and rodents have demonstrated that prolonged exposure to moderate-intensity noise (∼70–80 dB SPL) has no apparent effect on behavioral and auditory brainstem response (ABR) thresholds (Canlon and Fransson 1995), but instead results in neuroplastic changes throughout the auditory pathway (Pienkowski and Eggermont 2010; Pienkowski and Eggermont 2012; Zhou and Merzenich 2012; Sheppard et al. 2017).

The auditory system of rodents becomes sensitive to environmental sounds at approximately postnatal day (P) 12, when it undergoes extensive refinement and develops into the structurally and functionally mature state in the several weeks following hearing onset (Geal-Dor et al. 1993; de Villers-Sidani et al. 2007). Hence, exposure to different types of noise during the early developmental stages may have profound and cumulative effects on hearing impairment, compared to adulthood (Zhang et al. 2008; Grecova et al. 2009). Adult rats transiently deafened at P14, using a short-term high-intensity noise exposure (125 dB SPL), exhibited a worsening of frequency discrimination, and alterations of structure and function in the peripheral and central auditory systems (Pierson and Snyder-Keller 1994; Rybalko et al. 2015; Suta et al. 2015). Deprivation of acoustic experiences by rearing infant rats under conditions of continuous moderate-intensity white noise (65–70 dB SPL) from the early postnatal stage resulted in poorly developed cortical frequency receptive field structure and tonotopicity in the primary auditory (A1) cortex of rats, which may be attributed to altered expression levels of neurotransmitter receptors (Chang and Merzenich 2003; Xu et al. 2010, 2010a). The present study aimed to verify whether long-term moderate-level noise exposure during the critical developmental period affects the hearing phenotype of rats, and whether this gives rise to neural plasticity in the auditory cortex.

## 2. Materials and methods

### 2.1. Noise exposure

Sprague–Dawley rats (pregnant and newborn male rats, obtained from SLAC Laboratory Animal Co. Ltd, Shanghai, China) were used in the study. The animals were maintained under a 12/12 h light/dark circle, with access to standard food and water *ad libitum*. The white noise signal was produced using a white noise generator and amplified to 70 dB SPL that was measured near the cage. Rats in the noise exposure (NE) group were continuously exposed to this moderate-intensity white noise from P12 (8:00 am) to P30 (8:00 pm). Control group rats were maintained in an enclosed room with a normal sound environment (with background noise level at approximately 40 dB SPL) from P12 (8:00 am) to P30 (8:00 pm). The mothers were taken away from their male offspring on P21. All experimental procedures described here were carried out in accordance with the National Institutes of Health (NIH) guidelines for the Care and Use of Laboratory Animals and approved by the Ethics Committee and the Committee of Animal Experimentation of Shanghai University. All efforts were made to minimize the number of animals used and their suffering.

### 2.2. Auditory brainstem responses

Measurements of ABRs were carried out inside a sound attenuating booth, with a background sound level of approximately 30 dB (Industrial Acoustics Corp.). Control and NE rats (at 8:00 am on P31) were anesthetized with chloral hydrate (450 mg/kg, i.p.) and then placed onto a heating pad to maintain body temperature at 37°C. Subdermal needle electrodes (Rochester Electro-Medical, Inc.) were placed at the vertex (active, noninverting), the infra-auricular mastoid region (reference, inverting), and the neck region (ground). The acoustic stimuli for ABRs were produced and the responses recorded using a TDT3 system, controlled using BioSig software (Tucker-Davis Technologies, Inc.; TDT). Differentially recorded scalp potentials were bandpass filtered between 0.05 and 3 kHz over a 15 ms period. A total of 400 trials were averaged for each waveform, for each stimulus condition. The ABRs were elicited with digitally generated (SigGen; TDT) pure tone pips presented free field, via a speaker (TDT; Part FF1 2021) positioned 10 cm from the vertex. Symmetrically shaped tone bursts were 3 ms long (1 ms raised cosine on/off ramps and 1 ms plateau) and were delivered at a rate of approximately 20 per second. Stimuli were presented at frequencies of 4, 5.6, 8, 11.3, 16, and 22.6 kHz, and in 5 dB decrements of sound intensity from 90 dB SPL. The ABR threshold was defined as the lowest intensity capable of evoking a reproducible, visually detectable response. Amplitudes (μV) and latencies (ms) of the two initial ABR peaks (waves I, II) were then determined at 6 kHz. The analysis was carried out offline in BioSig on traces with visible peaks by setting cursors at the maxima and minima (trough) of the peaks. Latency was determined as the time from the onset of the stimulus to the peak, and amplitude was measured by taking the mean of the △V of the upward and downward slopes of the peak.

### 2.3. Local field potential recording

Control and NE rats (at 8:00 am on P31, separate groups from the ABR recording) were anesthetized with chloral hydrate (10%, 4.5 mL/kg, i.p.) and ethyl carbamate (20%, 2 mL/kg, i.p.), placed in a stereotaxic frame and implanted with a 16-channel nickel-chromium microelectrode array (impedance less than 1 MΩ). Electrodes were placed in the middle layer of A1 (6.3 mm posterior to bregma, 6.3 mm lateral to midline, 0.8 mm below the brain surface) according to rat brain topography. Recordings of the local field potential (LFP) were acquired in the absence of acoustic stimuli. To reduce various interferences of ambient electromagnetic fields, we placed the recording chamber in a Faraday cage. The LFPs were acquired as broadband signals (0.1 Hz–5 kHz) using an OmniPlex System (Plexon Inc., USA). Brains were sliced and stained with toluidine blue after recording to ensure that the electrodes were located in the correct position. The following data analysis steps were performed off-line with custom written MATLAB scripts. After the data were imported into the MATLAB environment, a random 10 s period in each recording was selected and extracted to create a single file. The LFP recordings were low-pass filtered with a cutoff at 300 Hz. Line noise artifacts were removed using a 50 Hz Butterworth notch filter. Power spectral density (PSD) was computed using the Welch technique, with Hamming windowing and a fast Fourier transform segment length of 512 samples, with a 256-sample overlap. Changes in power were analyzed for five frequency oscillations (*δ*: ∼1–4 Hz, θ: ∼4–8 Hz, *α*: ∼8–13 Hz, *β*: ∼13–30 Hz, *γ*: ∼30–90 Hz). Wavelet packet decomposition was used to extract these five frequency bands. These oscillations were chosen because preliminary analyses showed that specific spectral changes occurred in these frequency bands when animals were anesthetized. The power of each oscillation was computed separately.

### 2.4. Real-time quantitative PCR (qPCR)

Total RNA was extracted from A1 cortex samples of control (*n* = 12) and NE (*n* = 10) rats (at 8:00 am on P31) using Trizol (Sangong Biotech, China). The RNA integrity was confirmed with the Agilent 2100 Bioanalyzer (Agilent, USA) with clear characteristic peaks at 28S and 18S. First-strand cDNA was synthesized using PrimeScript RT Master Mix (Takara, Japan), and qPCR was performed using SYBR Premix Ex Taq^TM^ (Takara, Japan) and a CFX96 Touch™ Real-Time PCR Detection System (Bio-Rad, USA). Primers were synthesized by Invitrogen and listed in Table S1. Relative expression levels for the glutamate receptor transcripts were calculated by the 2^-ΔΔCT^ method. The expression of the glutamate receptors (GluRs) was normalized using *β*-actin and GAPDH as endogenous controls. Each experiment was repeated four to six times, with three independent RNA samples. The ‘n’ value represents the number of normalized values.

### 2.5. Immunohistochemical staining

Control and NE rats (at 8:00 am on P31) were deeply anesthetized and perfused with sterile saline and 4% paraformaldehyde in 0.1 M phosphate buffer (PBS; pH 7.4). Brains were removed and fixed using 4% paraformaldehyde for two days, then placed in 20% and 30% sucrose for dehydration. Serial coronal sections (20 μm) were collected using a freezing microtome and mounted on glass slides. Brain samples were washed three times with PBS for 5 min each and incubated in 0.5% Triton X-100 containing 3% H_2_O_2_, for 30 min at room temperature. After washing with PBS, samples were antigen-retrieved for 25 min with pepsin at 37°C. Next, samples were washed three times and blocked for 1 h with 5% goat albumin serum at room temperature. Primary antibodies against GluR1 (Santa Cruz, USA; 1:50), GluR2 (Abcam, UK; 1:200), NR1 (Santa Cruz; 1:50), NR2A (Santa Cruz; 1:50), and NR2B (Abcam; 1:200) were diluted in 5% goat serum. After incubation at 4°C for 24 h, samples were washed three times in PBS and then incubated with biotin-conjugated goat anti-rabbit immunoglobulin G (Weiao Biotech Co. Ltd, China) at room temperature for 30 min. Sections were again washed, then developed using the chromagen 3, 3’-diaminobenzidene, 5% diluted with PBS and 0.1% H_2_O_2_, for 8 min. Samples were dehydrated, cover-slipped and photographed using an upright microscope (Nikon, Japan). The A1 cortex (layer I-VI) of each slice was chosen according to the rat brain topography. The average optical density (Integrated Optical Density (IOD)/area) of three randomly selected, non-overlapping fields (300 μm × 200 μm) at × 200 magnification was assessed using Image-Pro Plus software (Version 6.0). One slice of A1 cortex from each individual rat was selected for one GluR subunit staining. The ‘n’ described in the results represents the number of A1 slices used for data collection in each experiment.

### 2.6. Statistical analysis

All data are presented as mean ± SEM and were analyzed with SPSS and GraphPad Prism software. The investigators who performed the data acquisition and quantification were blind to the experimental conditions. The differences between the control and NE groups were compared using two-way analysis of variance (ANOVA) followed by post hoc Scheffé test and unpaired Student’s two-tailed *t*-test. *P* < 0.05 was considered to be statistically significant.

## 3. Results

### 3.1. Early noise exposure impairs hearing sensitivity of rats

**Figure 1.**
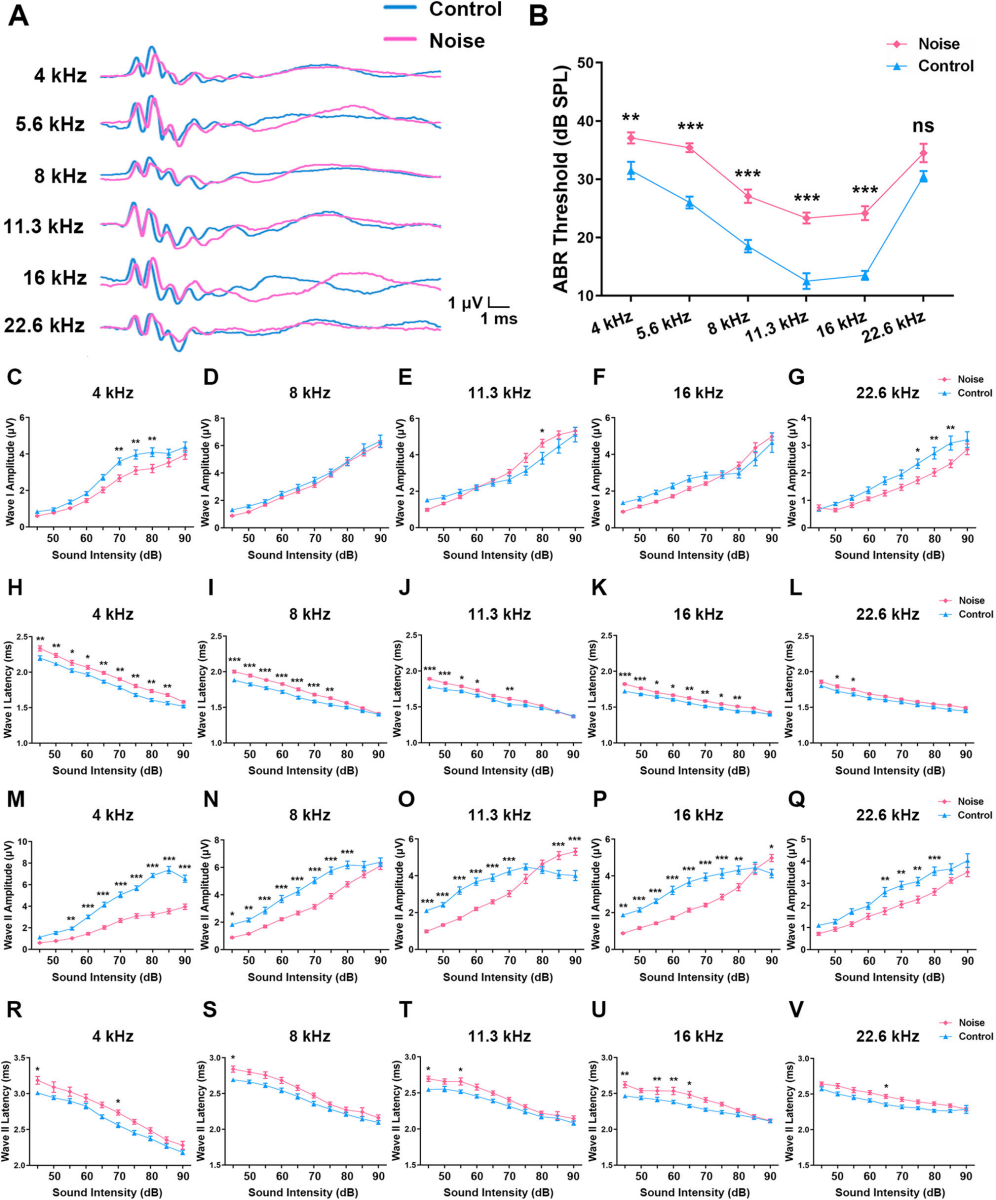
Changes in ABR parameters for control and NE rats. (a) A schematic of representative ABR waveforms (obtained at 80 dB) in control (blue line) and NE (red line) rats. (b) The graphs illustrate hearing thresholds (dB SPL) defined by measuring ABRs in control and NE rats at six sound frequencies (4, 5.6, 8, 11.3, 16, and 22.6 kHz). (c–g) Amplitude of wave I changes at five sound frequencies (4, 8, 11.3, 16, and 22.6 kHz, respectively) in control and NE rats. (h–l) Latency of wave I changes at five sound frequencies (4, 8, 11.3, 16, and 22.6 kHz, respectively) in control and NE rats. (m–q) Amplitude of wave II changes at five sound frequencies (4, 8, 11.3, 16, and 22.6 kHz, respectively) in control and NE rats. (r–v) Latency of wave II changes at five sound frequencies (4, 8, 11.3, 16, and 22.6 kHz, respectively) in control and NE rats. Control: *n* = 11 rats, NE: *n* = 13 rats. Data are shown as the mean ± SEM. Two-way ANOVA followed by a Scheffé post-hoc test was used for multiple comparison of ABR parameters of the control and NE rats, *P < 0.05, **P < 0.01, ***P < 0.001.

To explore whether prolonged moderate-level noise exposure during the developmental period affects hearing function, ABRs were measured to determine the hearing phenotype of NE rats (*n* = 13) and age-matched control rats (*n* = 11). Compared with controls, it was found that NE rats had elevated hearing thresholds ranging from low to high frequencies (4–16 kHz), but with no significant difference at the 22.6 kHz frequency (Figure 1(a and b)). The wave I amplitudes of NE rats were decreased between 70 and 85 dB SPL at 4 and 22.6 kHz, but increased at 80 dB SPL at 11.3 kHz (Figure 1(c–g)). The wave I latencies of NE rats were profoundly delayed at the majority of sound levels over the 4–22.6 kHz range (Figure 1(h–l)). In addition, the wave II amplitudes of NE rats were decreased in a large proportion of sound levels at low frequency (4 kHz), and decreased up to 80 dB SPL across the moderate and high frequencies (8–22.6 kHz) (Figure 1(m–q)). It was noted that at the high frequencies (11.3 and 16 kHz) the wave II amplitudes of NE rats were increased at 85–90 dB SPL (Figure 1(o and p)). Finally, the wave II latencies of NE rats exhibited a delay at several select sound levels across all frequencies (Figure 1(r and v)). Thus, analyses of the ABR waveforms reveal hearing impairment in rats after early NE, together with profound changes in the integrity of the auditory periphery and brainstem pathways.

### 3.2. Early noise exposure modifies LFP oscillations in the A1 cortex of rats

**Figure 2.**
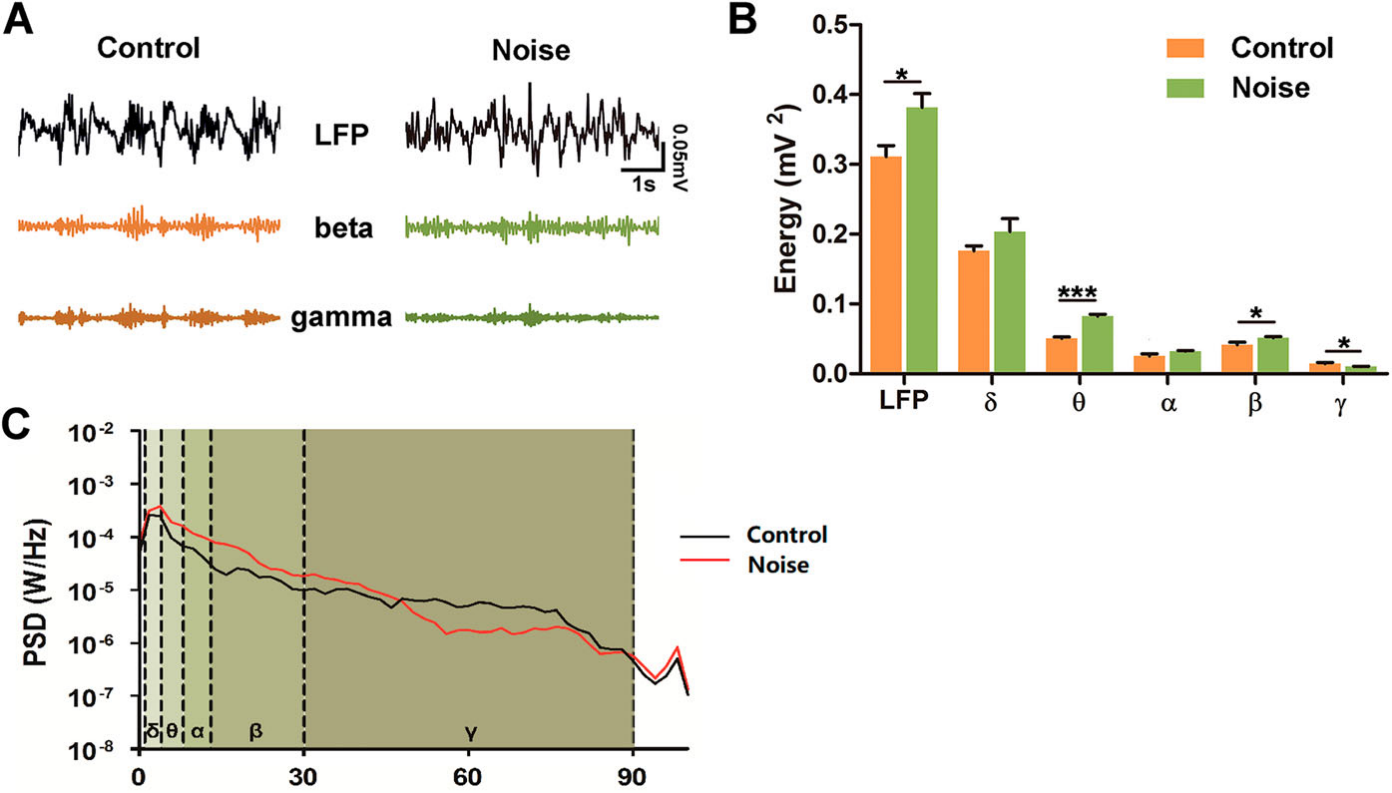
Changes in LFP characteristics in the A1 cortex for control and NE rats. (a) Random LFP segments recorded in the A1 of control and NE rats alone, and with *β* and *γ* oscillations extracted from these segments. (b) The power of five oscillations of LFP from the A1 cortex of control and NE rats is shown in the bar graph (mV^2^). (c) Average PSD from control and NE rats is shown after it was normalized and computed with fast Fourier transform (FFT). Each line chart was painted into five areas in order to distinguish one oscillation from the other. Control: *n* = 6 rats, NE: *n* = 6 rats. Data are shown as mean ± SEM. Asterisks indicate levels of significance determined by unpaired Student’s *t*-test: *P < 0.05, **P < 0.01 and ***P < 0.001.

To investigate whether changes in the auditory periphery following early NE induce central neuroplasticity, LFP oscillations in the A1 cortex were analyzed. The results showed that the raw LFP traces in the A1 cortex of NE rats were altered (Figure 2(a)). After the raw traces were extracted into five frequency bands, the PSD was found to be embellished across different frequency bands (Figure 2(c)). The total power of raw LFP oscillations in the A1 cortex was remarkably increased (by 22.68%, *p* < 0.05) in the NE rats, compared to control rats (control, *n* = 6; NE, *n* = 6). The power of the high-frequency *β* oscillation was enhanced (by 24.15%, *p* < 0.05), whereas that of the *γ* oscillation was reduced (by 28.47%, *p* < 0.05). The remaining lower-frequency θ oscillation demonstrated a remarkable power increase (by 62.78%, *p* < 0.001) (Figure 2(b)). These results indicate that early NE increased neural excitability in the A1 cortex of rats.

### 3.3. Alteration of GluR expression in the A1 cortex after early noise exposure

**Figure 3.**
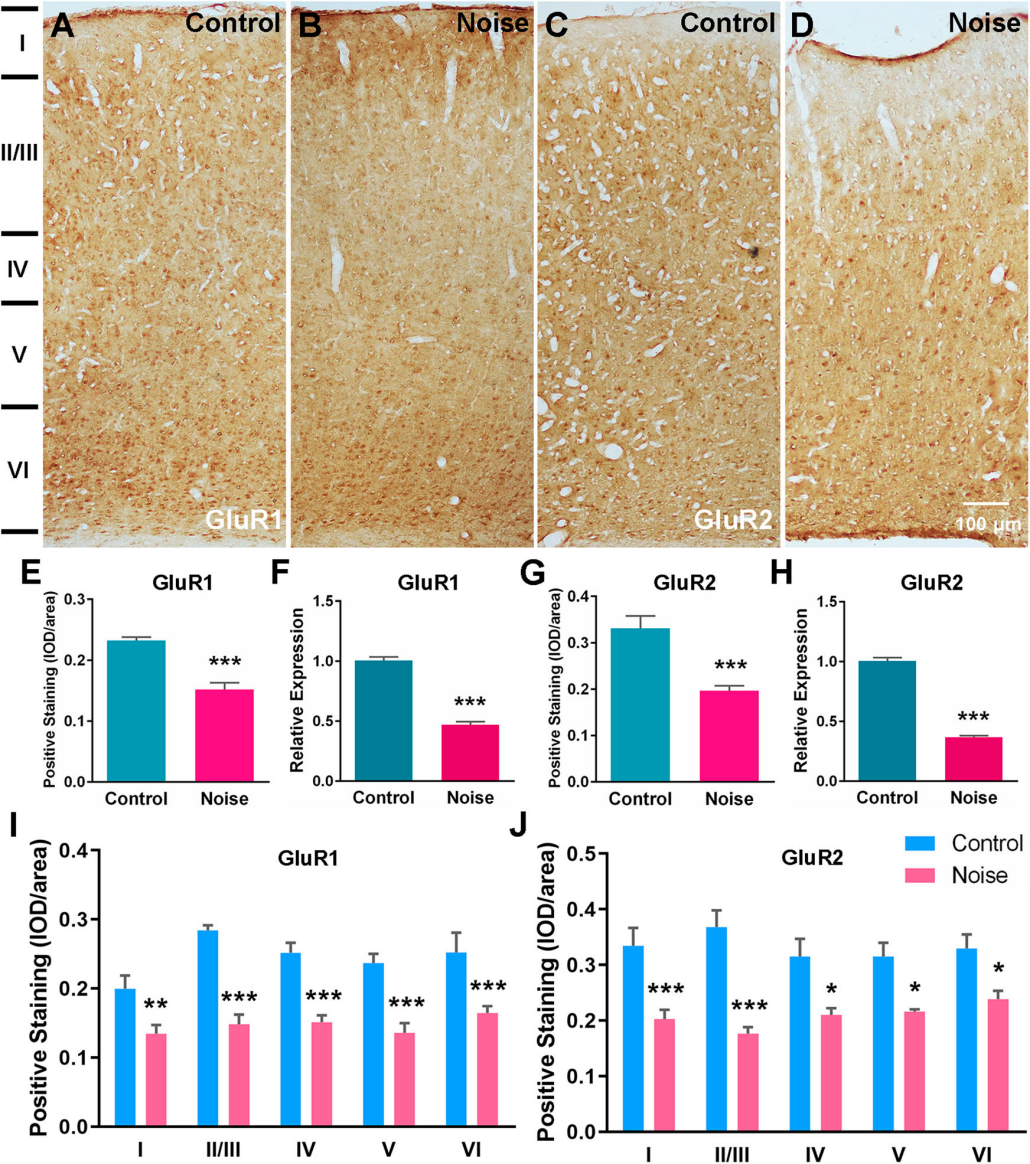
Downregulated expression of AMPA receptor subunits in the A1 cortex after NE. (a–d) Example coronal sections of the A1 cortex from control and NE rats stained for GluR1 and GluR2. (f, h) The mRNA levels of GluR1 and GluR2 from control and NE rats. All values were normalized against the mean of GAPDH. (e, g) The protein levels of GluR1 and GluR2 from control and NE rats. Data are presented with average optical density (IOD/area). (i, j) Changes in GluR1 and GluR2 levels in the layers (I, II/III, IV, V, VI) of the A1 cortex from control and NE rats. Data are presented as mean ± SEM. Unpaired Student’s *t*-test was performed to compare the differences of total protein and mRNA expression levels of the control and NE rats. Two-way ANOVA followed by a Scheffé post-hoc test was used for multiple comparison of different A1 cortical layers across two groups, *P < 0.05, **P < 0.01, ***P < 0.001.

**Figure 4.**
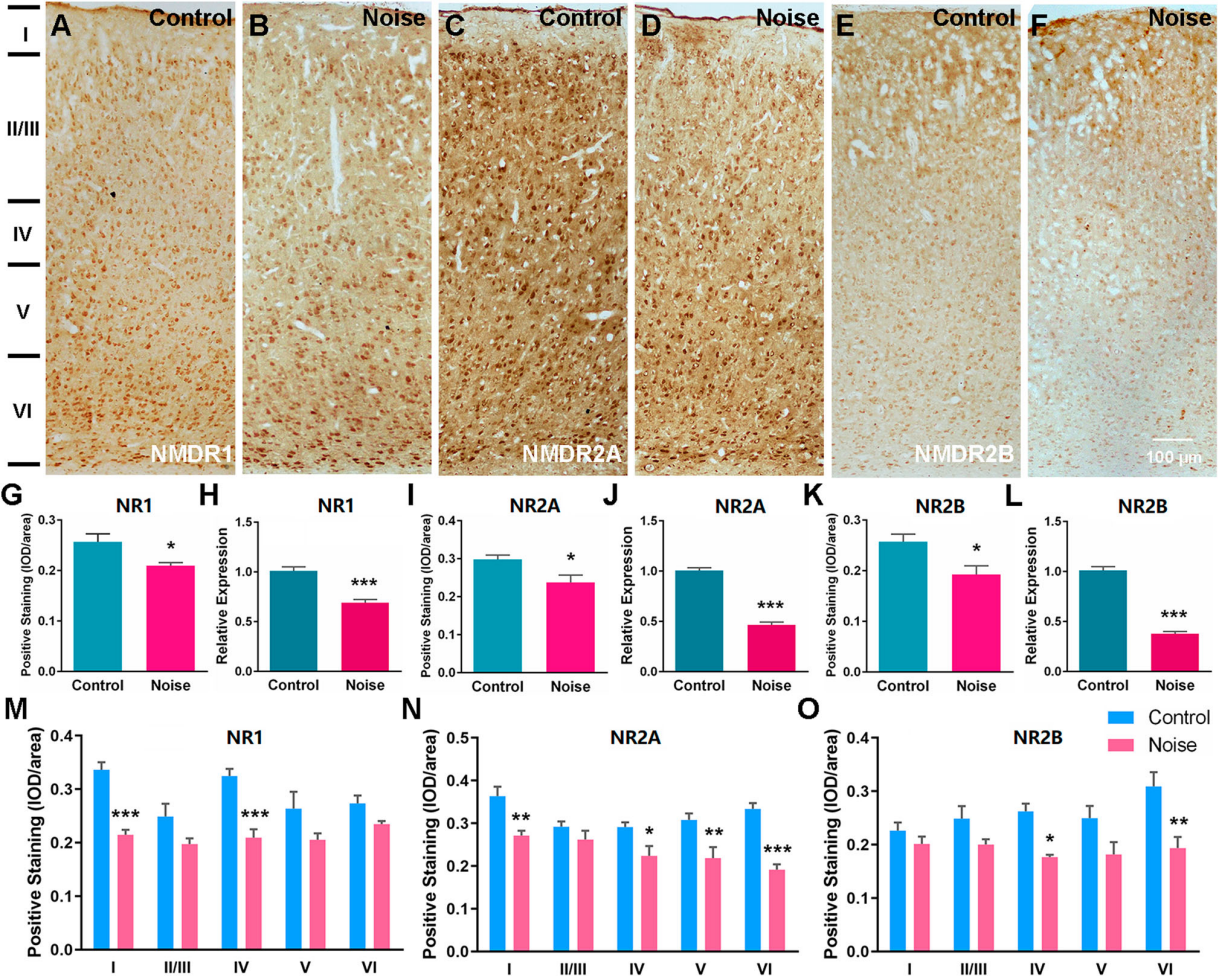
Downregulated expression of NMDA receptor subunits in the A1 cortex after NE. (a–f) Example coronal sections of the A1 cortex from control and NE rats stained for NR1, NR2A and NR2B. (h, j, l) The mRNA levels of NR1, NR2A and NR2B from control and NE rats. All values were normalized against the mean of GAPDH. (g, i, k) The protein levels of NR1, NR2A and NR2B from control and NE rats. Data are presented with average optical density (IOD/area). (m, n, o) Changes in NR1, NR2A and NR2B levels in the layers (I, II/III, IV, V, VI) of the A1 cortex from control and NE rats. Data are presented as mean ± SEM. Unpaired Student’s *t*-test was performed to compare the differences of total protein and mRNA expression levels of the control and NE rats. Two-way ANOVA followed by a Scheffé post-hoc test was used for multiple comparison of different A1 cortical layers across two groups, *P < 0.05, **P < 0.01, ***P < 0.001.

To pinpoint molecular clues underlying the changed neural excitability in the A1 cortex by early NE, the expression levels of two AMPA receptor (AMPAR) subunits (GluR1 and GluR2) and three NMDA receptor (NMDAR) subunits (NR1, NR2A, and NR2B) were analyzed. The qPCR experiments showed that the mRNA levels were decreased for both GluR1 (*p* < 0.001; control *n* = 14; NE, *n* = 15) and GluR2 (*p* < 0.001; control *n* = 16; NE, *n* = 12) in the A1 cortex of NE rats (Figure 3(f and h)). Accordingly, the protein levels were also decreased for GluR1 (*p* < 0.001; control, *n* = 5; NE, *n* = 5) and GluR2 (*p* < 0.001; control *n* = 6; NE, *n* = 6) as revealed by immunohistochemical staining (Figure 3(a–d, e and g)). Using laminar-analysis, GluR1 was found to be downregulated in layers I, II/III, IV, V, and VI of the A1 cortex (*p* < 0.01, *p* < 0.001, *p* < 0.001, *p* < 0.001, and *p* < 0.001, respectively), while GluR2 expression was also shown to be downregulated in these layers (*p* < 0.001, *p* < 0.001, *p* < 0.05, *p* < 0.05, and *p* < 0.05, respectively) (Figure 3(i and j)). In addition, the A1 cortex of NE rats displayed decreased mRNA expression for NR1 (*p* < 0.001; control, *n* = 15; NE, *n* = 16), NR2A (*p* < 0.001; control, *n* = 14; NE, *n* = 11), and NR2B (*p* < 0.001; control, *n* = 15; NE, *n* = 14) (Figure 4(h, j, l)). The protein levels were also decreased for NR1 (*p* < 0.05; control, *n* = 6; NE, *n* = 6), NR2A (*p* < 0.05; control, *n* = 6; NE, *n* = 6), and NR2B (*p* < 0.05; Control, *n* = 5; NE, *n* = 4) (Figure 4(a–f, g, i, k)). Downregulation of NR1 was found in layers I and IV (*p* < 0.001 and *p* < 0.001, respectively), downregulation of NR2A was seen in layers I, IV, V, and VI (*p* < 0.01, *p* < 0.05, *p* < 0.01, and *p* < 0.001, respectivly), and NR2B was downregulated in layers IV, and VI (*p* < 0.05, and *p* < 0.01, respectively) (Figure 4(m–o)).

## 4. Discussion

There is growing evidence that maturation of the auditory system depends on afferent activity supplying input to the developing centers, and that the structure and function of the auditory system may be severely affected by an unnatural acoustic environment during early ontogeny (Bures et al. 2017). The ABR, which consists of acoustically stimulated signals that represent the synchronized neural activation along the auditory ascending pathways (Melcher and Kiang 1996), has been widely used over the last decade with the hope of finding possible abnormalities related to hearing pathology. In the current study, we found that infant rats exposed to noise at a 70 dB SPL from the age of P12 to P30 experienced elevated hearing thresholds for low (4 kHz) to high (16 kHz) frequencies, which indicated that the prolonged moderate-level noise exposure during the critical developmental period widely impaired hearing level. Interestingly, this kind of noise exposure had no obvious effect on hearing threshold at the higher frequency (22.6 kHz), which hinted that the characteristic unnatural sound in the present study altered hearing function at selective frequencies. Furthermore, it was found that the wave I amplitudes of NE rats were selectively decreased at some sound frequencies and intensities, and their latencies were slightly delayed across all frequencies. These results strongly suggest that early noise exposure gives rise to profound changes in the integrity of the rat auditory periphery. On the other hand, the wave II latencies of NE rats were slightly delayed at some sound levels across all frequencies, indicating that the timing of synaptic transmission and nerve conduction in the cochlear nucleus may be impaired by early NE. Furthermore, the wave II amplitude in NE rats tended to be markedly lower than in control rats at the majority of sound intensities across all frequencies, suggesting that the number of activated neurons is decreased, and synchrony of firing is weakened, in the cochlear nucleus of NE juvenile rats. Most interesting, however, is the finding that under some sound conditions (85–90 dB, 11.3–16 kHz), the wave II amplitude became significantly higher in NE rats. There is growing evidence to suggest that the auditory system can compensate for peripheral loss induced by noise exposure through increased central neural activity – a phenomenon referred to as central gain (Robertson et al. 2013; Schrode et al. 2018). Thus, these findings led us to hypothesize that neural activity in the high-frequency area of the cochlear nucleus of NE rats could be enhanced to compensate for the peripheral loss. The results presented here indicate that long-term exposure to sound with moderate intensity during the developmental period of rats may result in a substantial impairment of auditory function, which can be observed both in the cochlea and brainstem.

The central auditory system is extremely plastic, capable of altering structure and function in multiple nuclei along the auditory pathway following changes in the peripheral system (Sheppard et al. 2014). Does the long-term moderate-level noise exposure during the developmental period lead to neural plasticity in the A1 cortex? It is well established that LFP oscillations are accompanied by the synchronization of activity within a widespread cerebral area, believed to be a common mechanism underlying neuronal assembly formation (David et al. 2009). In the present study, the enhanced power of raw LFP oscillations in the A1 cortex by early noise exposure indicated that moderate intensity noise exposure during the developmental stage could reinforce neural excitability in the A1 cortex. These results were in accordance with a previous study showing that rats exposed to continuous noise (approximately 80 dB from P5 to P50) exhibited greater long-term potentiation in the A1 cortex than controls reared in normal acoustic environments (Speechley et al. 2007). However, exposure of adult rats to a moderate level of noise did not have a significant effect on the cortical representation of sound frequency (Zhang et al. 2002). These results indicated that modification of the auditory system by moderate level noise exposure is dependent on the developmental time window. From the ABR and LFP data together, it could be concluded that the early NE gives rise to hyperactivity in the A1 cortex, to adjust for changes in the neural output from the peripheral auditory system.

The *γ* oscillation is the most favored rhythm in the auditory cortex and has been implicated in the coding of complex acoustic features (Vianney-Rodrigues et al. 2011). In the present study, the power of the *γ* oscillation in the A1 cortex was clearly reduced after early noise exposure. It appears that long-term noise exposure during the developmental stage may weaken the coding and integration of auditory information in the A1 cortex. The *β* oscillation in the auditory cortex may play a vital role in auditory–motor communication, reflecting a translation of timing information to auditory–motor coordination (Fujioka et al. 2009; Fujioka et al. 2015). The power of the *β* oscillation in the A1 cortex was significantly enhanced after early noise exposure, which suggested that moderate-intensity white noise exposure during the developmental stage may influence auditory–motor integration. Furthermore, it was found that the lower-frequency θ oscillation also underwent remarkable changes in the A1 cortex after early noise exposure. Recent studies have demonstrated that the lower-frequency oscillations are associated with predictive sensory processing (Arnal et al. 2015; Zhou et al. 2017; Pan et al. 2018). Further investigation is required to determine whether the changes in lower-frequency oscillations in the A1 cortex were involved in auditory and non-auditory adverse effects induced by moderate intensity noise during the developmental stage.

Synaptic transmission is the fundamental process underlying the conduction of neural information, and is important in neural plasticity. The AMPARs and NMDARs present at the majority of excitatory synapses in the central nervous system are important regulatory factors of synaptic plasticity (Traynelis et al. 2010), which may be profoundly modulated by environment and experience (Quinlan et al. 1999; Haas et al. 2006; Cai et al. 2010). In the present study, it was found that the expression levels of the AMPAR subunit GluR2 were significantly decreased in the A1 cortex (from layer I to VI) after early moderate-intensity white noise exposure, which is in keeping with a previous study showing downregulation of GluR2 expression in the A1 cortex following continuous moderate-level noise exposure during the developmental stage (P7 to P28) (Xu et al. 2010b). Furthermore, the expression level of GluR1, another important AMPAR subunit, was also observed to be downregulated in the A1 cortex (from layer I to VI) of NE rats, in the present study. These results strongly suggest that there is a drop-off of AMPA-mediated excitatory postsynaptic currents in the A1 cortex after prolonged NE. In addition to AMPARs, the expression levels of NMDAR subunits, including NR1, NR2A, and NR2B, in the A1 cortex were also found to be notably decreased with laminar specificity following noise exposure. However, this result is inconsistent with a previous study showing that early continuous noise exposure (65–70 dB SPL, from P7 to P56) has no effect on the expression levels of NMDA receptors (Xu et al. 2010). These discrepant results could be attributed to the different experimental strategies employed in the two studies. Nevertheless, the potential expression changes of NMDARs suggested that moderate-intensity white noise exposure could impair synaptic strength and long-term potentiation in different layers of the A1 cortex during the developmental stage.

Interestingly, the downregulation of GluR subunit expression seems to be incompatible with the enhancement of neural excitability in the A1 cortex after noise exposure. A previous study has revealed that continuous moderate-level noise exposure induces the downregulation of GABAA*α*1 and GAD65, and the upregulation of GABAA*α*3 in the A1 cortex, which suggests that noise rearing has powerful adverse effects on the maturation of cortical GABAergic inhibition (Xu et al. 2010). These data imply that glutamatergic excitation, as well as GABAergic inhibition, may be involved in the delayed maturation of the auditory receptive field structure and topographic organization of A1 after early noise exposure. Also, sensory cortices have a laminar architecture including six layers (I, II/III, IV, V, VI), which are believed to transform sensory information as excitation spreads serially along the layer IV→II/III→V/VI pathway (Constantinople and Bruno 2013). Layer V neurons comprise a major output of the cortex with the most substantial axonal innervation of subcortical and cortical structures, and layer VI neurons transmit feedback to the thalamus and cortex (Constantinople and Bruno 2013). It is noteworthy that the expression levels of three NMDAR subunits were found to be selectively downregulated in different layers of the A1 cortex in the current study, which indicates the possibility of a refined A1 intracircuit and efferent thalamocortical pathway in rats after continuous moderate-level noise exposure during the developmental period.

## 5. Conclusion

The present study showed that prolonged moderate-level noise exposure during the developmental period of rats can deteriorate the sound coding and transmission in the auditory periphery and brainstem ascending pathway, and subsequently modify oscillatory activities in the A1 cortex, accompanied by the alteration of expression of five excitatory receptor subunits. These findings may shed light on the cellular and molecular basis of noise-induced functional plasticity in the auditory system.

## Supplementary Material

Supplemental Material
