# Supplementary material for: Long-term exposure to moderate noise induces neural plasticity in the infant rat primary auditory cortex
Source: Anim Cells Syst (Seoul). 2019 Jul 19;23(4):260–9. doi: 10.1080/19768354.2019.1643782 (PMC6711034; doi:10.1080/19768354.2019.1643782)
Supplement: Supplemental Material [file TACS_A_1643782_SM7909.docx]

**Table S1. Sequence of primers for qPCR**

| **Name** | **Sequence（5’-3’）** | **Length (bp)** |
| --- | --- | --- |
| GluR1_F | ACAGAAGTCCAAGCCAGGTG | 131 |
| GluR1_R | ATTCGTAGGGGCTGAAACGG |  |
| GluR2_F | AAAAGACCAGTGCCCTCAGT | 140 |
| GluR2_R | TGCCACCTTCATTCGTTTCG |  |
| NR1_F | ACGTGTGGAGGAAGAACCTG | 188 |
| NR1_R | CGGCAGCACTGTGTCTTTTT |  |
| NR2A_F | CCGACATCCACGTTCTTCCA | 181 |
| NR2A_R | TATCCCAGCCCACAAAGCTG |  |
| NR2B_F | GCCCGACTAATTCCAAGGCT | 138 |
| NR2B_R | ATCGGCCCTTGTCTTTCAGG |  |
| β-actin_F | AGCACTGTGTTGGCATAGAGGTC | 152 |
| β-actin_R | ACTATCGGCAATGAGCGGTTCC |  |
| GAPDH_F | GCAAGTTCAACGGCACAGTCAAG | 126 |
| GAPDH_R | CGACATACTCAGCACCAGCATCAC |  |
